# Mammary gland stem cells and their application in breast cancer

**DOI:** 10.18632/oncotarget.12893

**Published:** 2016-10-25

**Authors:** Xing Yang, Hui Wang, Baowei Jiao

**Affiliations:** ^1^ State Key Laboratory of Genetic Resources and Evolution, Kunming Institute of Zoology, Chinese Academy of Sciences, Kunming, Yunnan, China; ^2^ University of Chinese Academy of Sciences, Beijing, China

**Keywords:** mammary gland stem cells, MaSCs, breast cancer stem cells, BCSCs, therapeutic perspectives

## Abstract

The mammary gland is an organ comprising two primary lineages, specifically the inner luminal and the outer myoepithelial cell layers. Mammary gland stem cells (MaSCs) are highly dynamic and self-renewing, and can give rise to these mammary gland lineages. The lineages are responsible for gland generation during puberty as well as expansion during pregnancy. In recent years, researchers have focused on understanding how MaSCs are regulated during mammary gland development and transformation of breast cancer. Here, we summarize the identification of MaSCs, and how they are regulated by the signaling transduction pathways, mammary gland microenvironment, and non-coding RNAs (ncRNAs). Moreover, we debate the evidence for their serving as the origin of breast cancer, and discuss the therapeutic perspectives of targeting breast cancer stem cells (BCSCs). In conclusion, a better understanding of the key regulators of MaSCs is crucial for the clinical treatment of breast cancer.

## MAMMARY GLAND BIOLOGY

The mammary gland produces and secretes milk to nourish offspring, and comprises a highly dynamic epithelial structure exhibiting in different development stages [1]. After birth, the mammary epithelia remains quiescent [2]. At puberty, however, the mammary gland expands considerably in response to hormonal cues and other factors to form a highly branched ductal network, a process that is referred to as ductal morphogenesis. In the mammary gland of virgin mice, for example, epithelial proliferation and apoptosis accompany each estrus cycle [3]. During gestation, however, the mammary gland expands further and the alveolar epithelium proliferates rapidly to develop secretory alveoli that are capable of producing milk. During lactation, directional luminal cells synthesize milk proteins and the secreting of oxytocin causes milk to move to the nipple through the branched ductal structure [2]. After lactation, the mammary gland ceases milk production, and the expanded epithelial compartment returns to the ‘resting’ state of puberty, known as involution [4].

The cycle of mammary gland development is controlled by the synergistic actions of hormones and growth factors, such as the ovarian steroid hormones estrogen and progesterone, and the pituitary growth hormone (GH) and prolactin. During puberty, ductal morphogenesis is driven prominently by estrogen, whereas progesterone activates side branching of the ducts during sexual maturity. Prolactin and progesterone initiate the formation of alveolar bud during gestation, as well as drive milk production during lactation [5]. In addition, estrogen receptors (ER) and progesterone receptors (PR) are critical for mammary gland morphogenesis. Loss of ER-α inhibits branching and elongation of mammary gland ducts, while the development of secretory alveoli is damaged in PR^−/−^ mutant mice [6, 7].

During the whole life of a female, the mammary gland undergoes constantly the cycles of proliferation, differentiation, and apoptosis, leading to the remodeling of the glandular tissue. Therefore, researchers suspected the existence of mammary stem cells (MaSCs) for many years, which were finally identified and isolated in mice in 2006 [8, 9]. MaSCs can self-renew as well as differentiate into different cells in mammary gland development. The self-renewal capacity of MaSCs should ensure and drive the growth and development of the mammary gland during its developing cycle. These features of MaSCs make them a vulnerable target of tumorigenesis. Thus, not only have the characteristics of MaSCs been studied in recent years, but their potential roles during tumorigenesis have also been intensely debated.

## MASCS AND PROGENITOR CELLS

**Figure 1:**
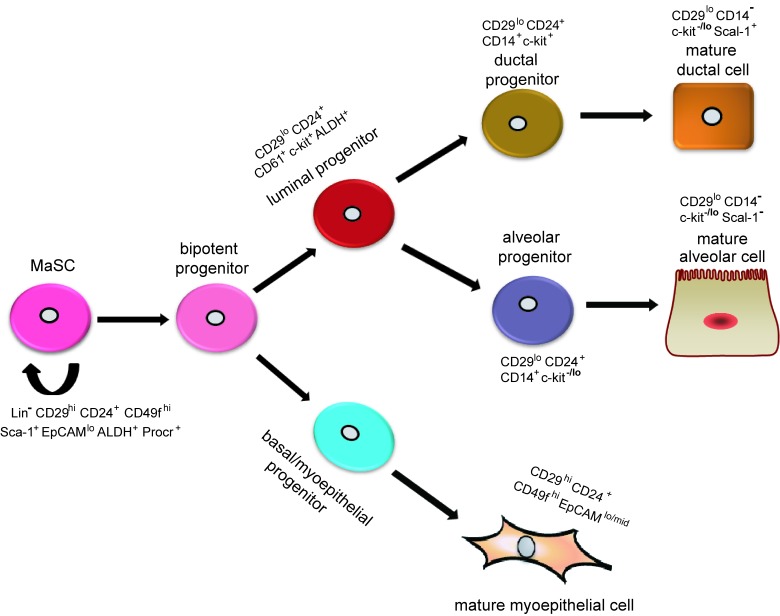
Hypothetical model of mammary epithelial hierarchy and markers of prospectively identified subsets in the mouse mammary gland A stem cell symmetrically or asymmetrically divides to generate a bipotent progenitor, which, in turn, gives rise to both luminal and basal/myoepithelial progenitor cells. Studies suggest that luminal progenitors differentiate restrictively to either ductal or alveolar cells. In contrast, basal/myoepithelial progenitors differentiate directly to basal/myoepithelial cells that are thought to be enriched for MaSCs. Currently, researchers use surface markers Lin, CD24, and CD29 to isolate basal (Lin^-^CD24^+^CD29^hi^) and luminal (Lin^-^CD24^+^CD29^lo^) cells. A specific marker for MaSCs remains unknown.

Adult mammary epithelial cells are composed of an inner luminal layer and outer myoepithelial/basal layer, which are thought to arise from a bipotent progenitor during embryonic development (Figure 1). Stem cells are capable of perpetuating themselves through self-renewal and have the potential to differentiate into all kinds of mature cells to form particular tissues [10]. Transplantation assays have suggested that MaSCs lead to the generation of the two mammary epithelial lineages - the luminal progenitor cells and basal cells [4, 11]. Luminal progenitor cells can be further subdivided into cells that are restricted to either ductal or alveolar cells. Basal cells consist of an enriched stem/progenitor cell population and myoepithelial cells, which are required for milk secretion during lactation [12].

MaSCs orchestrate the development of the mammary gland during embryogenesis. The identification and the isolation of MaSCs are important for determining their properties and functions. Due to a lack of specific MaSC markers, however, researchers initially utilized stem cell markers that are known in other organs to search for potential stem/progenitor cells in the mammary gland. For example, stem cell antigen1 (Sca-1), a marker of hematopoietic stem cells, was used to isolate mouse mammary gland stem/progenitor cells [13]. Shackleton and colleagues [8, 9] used CD29 (β1-integrin, a stem-cell marker in skin [14]) and CD24 (heat-stable antigen, a marker of neural stem cells [15]) to enrich MaSCs (Lin^-^CD29^high^CD24^+^). Ginestier et al. [16] suggested that aldehyde dehydrogenase1 (ALDH1) activity could provide a common marker for both normal and malignant mammary stem and progenitor cells. It has been reported that a combination of ALDH and Sca-1 can increase the specificity of progenitor populations in COMMA-D cells (murine mammary epithelial cell line) [17]. Recently, Wang et al. [18] identified that protein C receptor (Procr), marks a unique population of multipotent mouse MaSCs in mammary gland, which suggests that Procr^+^ cells are important for the development and maintenance of the adult mammary gland.

However, whether MaSCs are multipotent remains a controversial subject. Serial transplantation assays have indicated that a Lin^-^CD29^hi^CD24^+^ cell may reconstitute a complete mammary gland, which implies the single cells are multipotent and have the capacity of self-renewal and multi-lineage differentiation [9]. However, these assays usually do not show such differentiation under physiological conditions [19]. Conversely, genetic lineage-tracing experiments can mimic physiological conditions [20]. Using this approach, Van Keymeulen et al. [4] found that the expansion and maintenance of each basal and luminal cell was maintained by the existence of two types of long-lived and lineage-restricted unipotent stem cells, which could directly differentiate into either myoepithelial or luminal lineages, but not being maintained by rare multipotent stem cells. However, lineage tracing experiments do exhibit inherent limitations [21, 22], and thus, the existence of unipotent stem cells remains uncertain. In 2014, using a stochastic multicolor *cre* reporter combined with new three-dimensional imaging, researchers demonstrated the existence of bipotent MaSCs, and suggested that the unipotent stem cells described in previous studies might represent different progenitor cells [22]. In addition, the Zeng laboratory demonstrated that Procr represents a population of multipotent MaSCs, which are at the top of the mammary epithelial cell hierarchy [18], thereby sustaining that multipotent and unipotent stem cells co-exist in the mature mammary gland. Judging from the descriptions above, mammary epithelial cell hierarchy could be understood as follows: multipotent MaSCs give rise to bipotent stem cells, which differentiate into lineage-restricted progenitors and unipotent stem cells; lineage-restricted progenitors then differentiate into the myoepithelial and luminal epithelial lineages. How MaSCs give rise to progenitor cells or regenerate themselves are subjects to be further investigated. All in all, the purification and characterization of each mammary epithelial cell subpopulation provide an essential framework for defining the regulators and functions of MaSCs and progenitor cells (Figure 1).

The process of mammary gland development requires numerous factors to regulate the function of mammary stem cells at different stages. Knowledge on the mammary gland and MaSCs have significantly contributed to our understanding of mammary gland development and breast cancer. Here, we provide an overview of the regulatory mechanisms of MaSCs involved in mammary gland development and breast cancer.

## REGULATORY MECHANISMS FOR MASCS

The renewal and differentiation of MaSCs are strictly regulated by factors such as the signal transduction pathways, mammary gland microenvironments, and ncRNAs.

### Regulatory pathways of MaSCs

**Figure 2:**
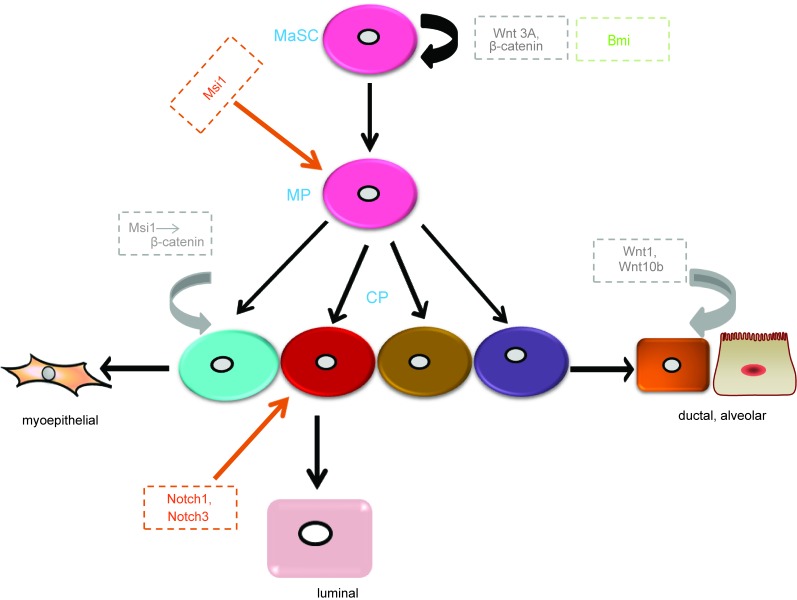
Main regulators of MaSCs in different signaling pathways Wnt3a regulates the maintenance and self-renewal of MaSCs. Wnt ligands, Wnt1 and Wnt10b, increase ductal branching and alveolar development. Msi1 increases progenitor expansion though increasing β-catenin. Notch1 and Notch3 increase the proliferation of luminal progenitors. Bmi maintains MaSC self-renewal though Shh signaling. MP, multipotent progenitor; CP, committed progenitor. Grey, Wnt signaling pathway; Orange, Notch signaling pathway; Green, Hh signaling pathway.

Once the regulatory pathways of MaSCs are destroyed or aberrantly regulated, cells will abnormally differentiate and proliferate, which could result in breast cancer. Wnt/β-catenin, Notch, and Hedgehog (Hh) signaling pathways are broadly involved in the regulation of MaSCs (Figure 2). However, the critical components of these pathways and how they influence mammary stem cell behavior remain unexplored.

Wnt signaling mediated-MaSCs have been described in numerous reviews [23–25]. Most importantly, intracellular Wnt is thought to act through canonical and noncanonical signaling pathways. The canonical Wnt signaling pathway, involving β-catenin as a key intermediate, is highly conserved in evolution and by far the best characterized of these pathways. The Wnt receptors are composed of Frizzled proteins together with one of the LDL receptor-related proteins (LRP5 or LRP6). Wnt ligands bind to their receptors and act *via* two cytoplasmic proteins, Disheveled and Axin, to inhibit the activity of a multi-protein complex that includes the tumor suppressor protein adenomatous polyposis coli (APC) and glycogen synthase kinase 3 β (GSK3β). The normal function of this complex is to phosphorylate β-catenin and thereby target it for ubiquitination and proteolysis. Once the activity of the APC-Axin complex is suppressed by Wnt signal transduction, β-catenin is accumulated in the cytosol and then translocated into the nucleus. Thereafter, β-catenin forms complexes with DNA-binding proteins of the Tcf/Lef1 family and stimulates the transcription of specific target genes [26]. The overexpression of *Wnt1* and *Wnt10b* leads to extreme branching and precocious alveolar development in virgin mice [27, 28], suggesting that Wnt/β-catenin signaling is essential for normal mammary gland development. Recently, studies have identified that Wnt/β-catenin signaling also promotes the proliferation and self-renewal of MaSCs [29]. Using an Axin2-lacZ reporter mouse model, previous research showed that adult mammary glands comprise a population enriched for stem cells, which is response to Wnt signalings. [29, 30]. Wnt3A greatly increases the clonogenicity of MaSCs. Furthermore, in long-term cell culture at the presence of Wnt3A, MaSCs can retain their self-renewal and differentiation ability *in vivo* [29]. In addition, constitutively activated β-catenin leads to excessive stem cell renewal/proliferation [30]. β-catenin has been indicated as a stem cell survival factor in the mammary gland [31]. Moreover, some proteins regulating the Wnt signaling pathway mediate the function of MaSCs. For instance, Msi1 is a homologue of the *Drosophila* Musashi protein and a neuroglial stem cell marker, and is expressed in the mouse mammary epithelial cell line and results in progenitor cell proliferation by increasing nuclear localization of β-catenin [32]. In summary, both Wnt signaling itself and its relevant proteins are involved in the regulation of MaSCs functions. Aberrant Wnt signaling through silencing of endogenous inhibitors or overexpression of Wnt ligands have been reported in human breast cancer [29, 33].

The Notch signaling pathway consists of ligands, receptors, and target genes. The Notch receptor has four homologs in mammals (Notch1-Notch4). The structure of the Notch receptor consists of a Notch extracellular structural domain (NEC) and transmembrane domain/Notch intracellular structural domain (NICD). All Notch ligands are single-stranded transmembrane proteins, named Delta-like 1, Delta-like 3, Delta-like 4, Jagged 1, and Jagged 2 [34–36]. Notch signaling transduction is initiated and activated by ligand binding, which involves proteolysis and endocytosis of the receptor. When the receptor is activated, it liberates the NICD, which is translocated into the nucleus and subsequently interacts with the DNA-binding protein CSL to induce the transcription of target genes [37, 38]. The expression of Notch ligands, receptors, and transcriptional targets are important regulators of mammary stem cells, luminal progenitors, and mature luminal enriched populations. In the mammary gland, Notch receptors are expressed in the luminal compartment. Here, we mainly focus on Notch1 and Notch3. By lineage tracing, Rodilla and colleagues [39] demonstrated that Notch1 is expressed in ERα^neg^ luminal progenitors and increases the self-renewal capacity of MaSCs. Notch3, a receptor associated with triple negative breast cancer [40], is involved in the maintenance of stem cells in other tissues [41–43]. Lafkas et al. [44] revealed that Notch3 is expressed in luminal progenitor cells. In addition, they found that proliferation of these cells was controlled by Notch3 activity using gain-of-function Notch3 mutant mice. However, knockdown of the Notch effector Cbf-1 in MaSC-enriched populations resulted in a significant increase in mammary repopulation capacity, demonstrating that downregulation of the pathway leads to MaSCs expansion. While, luminal progenitor cells expand and self-renew, eventually leading to the development of tumors in the presence of continual Notch signaling [45]. In addition, as mentioned above, overexpression of Msi1 leads to increased proliferation of progenitor cells; however, it maintains Notch activity and the transcription of downstream ligands and effector genes by inhibiting Numb (a cell fate determinant that interacts with Notch-1) [32]. Thus, another mechanism of Msi1 promoting mammary progenitor/stem cell proliferation could be the upregulation of Notch signaling. In summary, the Notch pathway plays a key role in MaSC expansion and luminal cell-fate commitment.

Hh signaling was first identified in *Drosophila*, where Hh is a segment polarity gene that regulates embryonic patterning [46]. It is an evolutionarily conserved system that controls patterning and cell fate from *Drosophila* to humans. The crucial components of the Hh signaling pathway are comprised of ligands (Sonic hedgehog, Shh; Indian hedgehog, Ihh; Deser hedgehog, Dhh), receptors (Patched-1 and -2 as well as Ptch1 and 2), effectors (Smoothened, Smo), and transcription factors (Gli1-3) [47]. In the absence of Hh ligand binding, the receptor Ptch localizes at the primary cilium and inhibits the co-receptor Smo, which is an essential positive mediator of the entire pathway, resulting in the phosphorylation and cleavage of transcription factors Gli2 and Gli3. These cleaved transcription factors act as repressors of target gene transcription. Once Hh binds to its receptor, Ptch no longer represses Smo and consequently Gli2 and Gli3 are not cleaved, thus activating Hh target genes [48]. Studies have described that Hh signaling is involved in the regulation of MaSCs. For instance, Ptch1, Gli1, and Gli2 are highly expressed in normal mammary stem/progenitor cells and are down regulated in differentiated cells [49]. Fiaschi et al. [50] reported that Gli1-induced tumors are involved in the expansion of epithelial cell populations that express putative progenitor cell marker cytokeratin 6. The Hh signaling pathway also targets other transcription factors in MaSCs. For example, the activation of the Hh signaling pathway increases the self-renewal and proliferation of MaSCs by stimulating transcription factor Gli and polycomb gene Bmi-1 [51]. Bmi1 is a downstream effector of Shh signaling [51], and its loss has an inhibitory effect on the proliferation and differentiation of MaSCs [52].

Other regulatory pathways of MaSCs include signal transducer and activator of transcription-5a and -5b (STAT5), which are both highly conserved. Although the STAT5 transcription factors are not important for MaSCs to reconstitute a functional mammary gland, Yamaji and colleagues [53] showed that the absence of STAT5 results in impairment of alveologenesis and lactation due to a reduced number of alveolar luminal progenitor cells in the virgin state. In addition, loss of STAT5 does not affect CD61^+^ luminal ductal progenitor populations, but does cause a decrease in CD61^+^ luminal alveolar progenitor cells [54]. The p53 pathway also plays a considerable role in the control of stem cell function in various tissues, including the mammary gland [55, 56]. Deletion of p53 enhances the self-renewal capacity of MaSCs *via* modulation of the Notch pathway [57], inhibition of rapid cell cycle progression [55], and prevention of epithelial-to-mesenchymal transition (EMT) program activation [55]. However, the precise molecular mechanism between p53 loss and stemness is unknown, which needs further investigation.

### Mammary gland microenvironment

Somatic stem cells are sustained and controlled by the surrounding microenvironment (niche), which is locally restricted to supporting the self-renewal of stem cells and preventing their differentiation. Similarly, MaSCs are also stably maintained within specific microenvironments. How does the microenvironment maintain tissular growth, cellular differentiation, and development of the mammary gland? As discussed below, a complex network exists among luminal cells, basal cells, stroma, and their microenvironments, which involves signaling from extracellular matrix (ECM) molecules, stromal-derived growth factors, and cytokines, and proteolytic enzyme activity in the microenvironment.

In mammary microenvironment, steroid hormones profoundly influence the behavior of MaSCs [21, 58], despite a lack of estrogen and progesterone receptors [21, 59]. Asselin-Labat and colleagues [21] found that ovariectomy markedly reduced MaSCs numbers *in vivo*, while treating with estrogen plus progesterone in mice, the activity of MaSC was increased [21]. Using the mouse estrus cycle as a model, Joshi et al. [58] demonstrated that alternation in MaSC numbers was associated with the estrus cycle, while progesterone was at maximal levels during diestrus. To examine the effect of progesterone on MaSCs, they also injected hormones to bilaterally ovariectomized mice and found that treatment with 17β-estradiol plus progesterone induced MaSC amplification [58]. In addition, Lombardi et al. [60] demonstrated that the GH receptor (GHR) was expressed in normal human mammary epithelia, and progestin treatment increased GH secretion, resulting in an increased number of cycling stem/progenitor cells.

Cytokines and growth factors are important mediators of MaSCs in the mammary gland microenvironment. Receptor activator of nuclear factor-κβ ligand (RANKL) has been indicated in mammary progenitor cell maintenance [61], and is a downstream effector of progesterone-mediating mammary lobuloalveologenesis [62]. Furthermore, progesterone stimulation has been shown to promote mammary epithelial proliferation by activation of RANKL in mice [21, 62]. Pregnancy leads to a hormonal environment, which influences the function of MaSCs. Although hormonal control is complex, progesterone has a prominent role in the establishment and maintenance of pregnancy. Asselin-Labat et al. [21] demonstrated that RANKL is a key mediator of MaSC function through paracrine in established pregnancy. In addition, Pellegrini et al. [61] also found that constitutive RANK expression breaks the balance between basal and luminal cells, leading to activation and expression of miR-146b in MaSCs and luminal progenitors. R-Spondin1 (Rspo1) is a potent WNT signaling enhancer and stem cell renewal mediator [63]. The inhibition of RANK signaling results in the activation of Rspo1, which suggests it is a key downstream effector of RANK on the functional regulation of mammary progenitors [64]. Similarly, Cai et al. [65] identified Rspo1, a novel hormonal mediator in the mammary gland, can promote MaSC self-renewal cooperated with Wnt4 through Wnt/β-catenin signaling. The transforming growth factor-β (TGF-β) superfamily has an important role in mammary gland development. Bone morphogenetic protein (BMP) is a soluble member of the TGF-β superfamily, and controls the function of stem cell regulation in many systems, including the mammary gland [66]. Chapellier et al. [67] demonstrated that BMP2 is an important regulatory factor of the stem cell niche, and controls the luminal differentiation of mammary progenitors. The ECM is a main regulator of epithelial function. In the mammary gland, myoepithelial cells exist in a specialized layer of the ECM, called the basement membrane (BM). Using microenvironmental protein microarrays, Studies have shown that ECM molecules influence mammary progenitor cell fate decisions [1, 68]. For example, laminin-1 inhibits the growth of mammary progenitor cells and maintains them in a quiescent state, whereas P-cadherin compels the differentiation of progenitor cells into myoepithelial cells [69]. Conversely, cell-cell connection, or expression of E-cadherin, facilitates progenitor cells differentiation into luminal epithelial cells [1]. Thus, the expression of the above microenvironmental proteins could mediate progenitor cell fate. Proteolytic actions remodel the ECM and stroma as well as release growth factors and cytokines. Thus, proteases are important for mammary gland development and function. The well-known enzymes in the condition of mammary gland development and differentiation are matrix metalloproteinases (MMPs), which are a family of extracellular zinc-dependent endopeptidases that contribute to a wide range of physiological and pathological processes [5, 6]. MMP3/stromelysin-1 is mostly produced by stromal fibroblasts and can promote epithelial-branching morphogenesis during puberty [7]. Using transplantation and mammosphere formation assays, researchers have also demonstrated that overexpression of MMP3 promotes MaSC self-renewal and differentiation [1]. In accordance with this, MMP3-deficient mutant mice reveals decreased numbers of MaSCs and diminished mammary-reconstituting activity [1].

### ncRNA regulation of MaSCs

Non-coding RNA is a kind of RNA molecule that is transcribed from the genome, but does not encode proteins. In recent years, ncRNAs have become an increasingly hot topic of research. Regulatory ncRNAs can be classified into two classes according to their length: small ncRNAs, which contain short (< 200 nt) RNA species, such as small-interfering RNAs (siRNAs), piwi-interacting RNAs (piPNAs), small nucleolar RNAs (snoRNAs), and microRNAs (miRNAs); and long ncRNAs (lncRNAs), which contain several types of transcripts 100s to 1000s of nucleotides long [70]. They participate in the regulation of all fundamental processes of development and tissue homeostasis, for instance, stem and progenitor cell regulation, cell-fate commitment, and differentiation. Next, we will focus on the role of ncRNAs (especially miRNAs and lncRNAs) in the regulation of MaSCs.

MicroRNA is a small ncRNA molecule and interacts with the 3′ untranslated regions (3′ UTRs) of targeting messenger RNAs to suppresses gene expression. They regulate numerous biological processes, including cell proliferation, stem cell maintenance and differentiation. The essential function of miRNAs in the various stages of mammary development is now recognized, and they have a main role in the regulation of developmental processes, proliferation, differentiation, and apoptosis. The expression of miRNAs in different cellular sub-populations has also been determined. For instance, miR-146b was found to be upregulated in basal cells and enriched in alveolar progenitor cells isolated from the mouse mammary epithelial cell line COMMA-1D [71–73]. In addition, miRNAs have also been described in the functional regulation of MaSCs. In limiting dilution transplantation experiments of primary mammary epithelial cells, passivation of the miR-193b locus, which targets STAT5 in mice mammary epithelia, resulted in elevated mammary stem/progenitor cell activity [74]. Using a small RNA library, Ibarra et al. [17] found that miR-205 and miR-22 were consistently enriched in the progenitor population, suggesting that they might be important for the identity of basal cells. For example, inhibition of miR-205 converts the epithelial phenotype to the mesenchymal phenotype (EMT) and promotes the stemness phenotype in mammary epithelial cells. Furthermore, miR-205 has also been implicated in the polarity of stem cell division and cell fate through concerted regulation of Zeb1 and Notch2 [75]. The overexpression of miR-22 in human or mouse mammary cells induces the upregulation of Zeb1/2, leading to a mesenchymal phenotype, expansion of the MaSCs, tumorigenesis, and metastasis [76]. Interestingly, miR-205 and miR-22 act as regulators of the EMT through regulation of the miR-200 family [76, 77], which are down regulated in normal MaSCs and breast cancer stem cells (BCSCs). Overexpression of miR-200c suppresses both clonogenicity of BCSCs and normal mammary outgrowth *in vivo* through targeting BMI1, a critical gene for self-renewal in many types of stem cells [78].

Long noncoding RNAs are rising as remarkable mediators of many important processes, such as the regulators of stem/progenitor cell functions, and as modulators of gene expression through different mechanisms at both the transcriptional and post-transcriptional level. As much as 38% of lncRNAs have been given to cooperate with various chromatin-modifying complexes and 24% specifically interact with polycomb repressive complex2 (PRC2) [79]. H19, one of the earliest identified regulatory lncRNAs, might have an influence on mammary gland development. H19 is reported to be induced by estrogen and enriched in terminal end buds (TEBs) in pubertal mice and in the alveolar cells of pregnant mice [80]. In addition to restricting growth during embryonic development, recent data have reported that H19 ensures the maintenance of long-term hematopoietic stem cells [81]. Similarly, if H19 plays a part in the mammary gland, it might sustain stem and/or progenitor populations during highly proliferative pubertal and pregnant stages of mammary development [82]. Additionally, H19 is up regulated in breast cancer, suggesting an oncogenic role [83], although the mechanism is still poorly understood. The effects of other lncRNAs on MaSCs and mammary gland development are less well studied. Only SRA, Zfas1 (Znfx1 antisense 1), and mPINC (mouse pregnancy-induced non-coding RNA) have been observed to have a regulatory function in mammary development [84–87]. Thus, the roles of lncRNAs on MaSCs, mammary development, and tumorigenesis need to be further investigated.

## BREAST CANCER AND MASCS

Breast cancer is a leading cause of death in women worldwide. Although breast cancer can be diagnosed early and better treatment has accompanied medical advances, its mortality rate remains high due to recurrence and metastasis [88]. Breast cancer is a heterogeneous disease. Heterogeneity of breast cancer is not only characterized by the same tumor type (intraheterogeneity), but also by diverse breast tumor subtypes (interheterogeneity) [89]. For interheterogeneity, breast cancer can be classified into different subtypes by histological and clinical factors. Eighteen different histological subtypes of breast cancer have been defined by the World Health Organization (WHO). In addition, molecular profiling also displays interheterogeneity of cancer [90]. These molecular profiling alterations will lead to the expression of oncogenes and the inhibition of tumor suppressor genes, which change the gene networks in normal mammary tissue. Based on molecular profiling alterations, breast cancer can be classified into five molecular subtypes, including luminal A, luminal B, HER2 positive (HER2^+^), basal-like, and normal-like [91–93]. Luminal A breast cancer expresses both estrogen receptors (ER^+^) and/or progesterone receptors (PR^+^/PR^-^), and is absent of HER2 expression. Luminal B is similar to luminal A, but with HER2 amplified. HER2^+^ breast cancer is characterized by HER2 expression and the absence of ER and PR. Basal-like subtypes negatively express ER, PR, and HER2. The gene expression signature of normal-like breast cancer is similar to that of normal mammary gland [91, 94, 95]. Different breast cancer subtypes show differences in survival rate, tumor incidence, and response to treatment. Positive outcomes have been observed in luminal tumors treated with hormonal therapy (tamoxifen). Because of high proliferation, HER2^+^ breast cancer always shows poorer outcomes, even when treated with HER2 antibodies such as trastuzumab [96]. Basal-like breast cancers are the most malignant cancers with poor patient outcomes and high levels of recurrence after treatment. Indeed, the complexity of this breast cancer is greater than that of previous subtypes. Recent research screened 2000 breast tumors and found a novel molecular classification of tumors, with ten diverse subtypes by combination of inherited and acquired genetic alterations [97]. For intraheterogeneity, breast cancer tissue includes different cell types and shows different morphological appearances at the histological level. This is mirrored by variable gene expression signatures in tumor tissues [98]. Currently, two different models show the origin of tumor heterogeneity: stem cell hierarchy and clonal evolution.

In the stem cell hierarchy model, cancer cells are considered to originate from cancer stem cells (CSCs) [99]. The theory of CSCs arises from the correlation between embryonic stem cell-induced teratocarcinomas and tumors [100]. Regarded as malignant stem cells, CSCs were first found in acute myeloid leukemia and are featured by stem cell-like characteristics, including self-renewal ability and differentiation potential [101]. CSCs divide into daughter cells that maintain the feature of self-renewal, while other daughter cells differentiate into neoplastic cells that form tumors. CSCs have been recognized in many kinds of solid tumor, including breast, prostate, brain, and lung cancer [102, 103]. BCSCs were the first to be reported in solid tumors. In breast cancer, tumors are hierarchically organized. The capability of self-renewal of BCSCs contributes to the growth, metastasis, and recurrence of breast tumor. In addition, the hierarchy model indicates that CSCs are derived from the transformation of normal stem cells, which means that a rare population of cells is more tumorigenic than a non-CSC population [102]. Although *in vitro* differentiation and *in vivo* xenograft data show that the human mammary gland is hierarchically organized, which supports the BCSC hypothesis [104], it is still elusive whether BCSCs derive from transformed progenitor cells, MaSCs, or both. Evidence from Al-Hajj et al*.* [105] proved the existence of BCSCs, and that a minority subpopulation of human breast cancer expressed the surface markers of CD44^+^ and CD24^−/low^ and could form heterogeneous tumors. Their ability to form tumors showed a 10- to 50-fold increase compared with other tumor breast cancer cells [105]. However, the existence and origin of CSCs remain the subject of skepticism and intense debate. Some researchers believe that CSCs do not necessarily arise from the transformation of normal stem cells, and thus, they prefer to name these cells as ‘cancer-propagating cells’ or ‘cancer-initiating cells’.

Currently, there are two hypotheses on the origin of BCSCs, that is, they arise either from MaSCs or from more differentiated and committed progenitor cells that acquire the ability of self-renewal *via* genetic and epigenetic reprogramming [96]. The mammary gland is a highly dynamic tissue. During pregnancy, MaSCs (CD49f^+^/CD29^+^/CD24^+^ repopulating cells in a mouse model) are influenced by hormones and serve as a source of proliferation and differentiation for different mammary structural units and for developing a milk-generating breast [8, 9]. As a result of the relatively long life span and ability of self-renewal, MaSCs have been advised as likely candidates for the initial malignant transforming events that drive cancer formation [106]. However, current clonal analyses and lineage-tracing experiments have identified that both luminal and myoepithelial progenitor cells are clonally expanding and maintain proliferation in adulthood [4], which means that these cells also are possible targets for tumor cellular transformation. Furthermore, other research has shown that luminal progenitor cells are a possible transformation target in basal-like breast cancers. Specifically, Lim et al. [59] firstly isolated and purified stem/progenitor cells (CD49f^high^EpCAM^-^; expressing p63/vimentin/CK14, but not ER/PR), luminal progenitor cells (CD49f^+^EpCAM^+^; expressing high levels of CK8/18/ER/GATA-3/MUC-1), and mature luminal cells (CD49f^-^EpCAM^+^; expressing high levels of ER/PR) from normal mammary gland preneoplastic samples from volunteers who were heterozygous for BRCA1 mutation. BRCA1 mutations are clinically associated with the development of basal-like breast cancers [107]. The results of Lim et al. [59] showed that CD49f^high^EpCAM^-^ basal stem cells were significantly reduced, while CD49f^+^EpCAM^+^ luminal progenitor cells were dramatically increased in the BRCA1-mutant samples. These observations, combined with succeeding gene expression profiling and functional studies, imply that a luminal progenitor population might be the transformation target in BRCA1-mutational basal breast tumors [59, 108]. Another mouse model of BRCA1-deficiency in either luminal progenitor cells or basal stem cells demonstrated that deletion of BRCA1 in the luminal progenitor cells, rather than the basal stem cells, phenotypically and histologically induced basal-like breast cancers [109]. In addition, it has been reported that luminal progenitor cells are the origin of TP53 mutated basal-like breast cancers [109, 110]. Although evidence supports that luminal progenitors are the cells-of-origin of basal-like breast cancers, there is no evidence showing that the malignant transformation of BCSCs does not originate from MaSCs. Actually, epidemiological studies have validated that an early and full-term pregnancy at a young age efficiently decreases the lifetime risk of breast cancer [111, 112]. Depletion of MaSCs used for differentiation during pregnancy might be the reason why early pregnancy is protective against breast cancer. Two recent reports have indicated that significant expansion of MaSCs during pregnancy form many tumor features [21, 113], suggesting that expansion and transformation of MaSCs could induce the formation of these tumors. Thus, the precise cells-of-origin of BCSCs need to be well defined in further studies. The ability to better trace MaSC populations *in vivo* and directly proof their susceptibility to transformation in particular forms of breast cancer are essential.

Most data on the organization of the human mammary gland are inferred from a combination of results from *in vitro* assay, xenotransplantation, and flow cytometry. Human MaSCs were previously thought to show a CD49f^+^/ESA^-/low^ phenotype, implying a basal location in the mammary gland [59, 114]. Actually, the precise location and hierarchy of human MaSCs are still elusive. It remains undecided whether MaSCs differentiate into a common bipotent progenitor giving rise to directed progenitors [108]. Therefore, the possible cause for the currently undetermined cells-of-origin of BCSCs is the lack of specific markers for such lineages. Generally, existing data indicate that human BCSCs are enriched in CD44^+^CD24^-/low^ [105], PKH26 [115], ALDH^+^ [16, 116], and SP [117, 118] dye-retaining cells. BCSCs were first isolated by cell-surface markers (CD44 and ESA), and without CD24 [105]. Furthermore, Lin^-^/ESA^+^/CD44^+^/CD24^-/low^ BCSCs are reportedly more tumorigenic than CD44^+^/CD24^+^/ESA^-^ cell populations. The molecular profiles of CD44^+^ and CD24^+^ cells show that CD44^+^ cells express basal stem cell markers, while CD24^+^ cells express markers of differentiated luminal cell [119]. Nevertheless, only a small population of CD44^+^/CD24^-/low^ cells are highly tumorigenic, which suggests that these markers can be applied to isolate and enrich BCSCs, while they might be not a pure CSC population [120, 121]. ALDH1 is considered a good BCSC marker and an independent predictor of poor outcomes in breast cancer patients [122, 123]. ALDH1A1 is one of main isotypes of ALDH1. Ginestier et al. [16] reported that ALDH1A1 mRNA levels are positively correlated with poor clinical outcomes, and that CD44^+^/CD24^-/low^/ALDH1^high^ BCSCs are more tumorigenic. Consistently, Morimoto et al. [124] also reported that ALDH1-positive breast cancers are more aggressive than other types of breast cancer. Charafe-Jauffret et al. [125] showed that ALDH1A1-positive breast cancer cells promote tumor invasion and metastasis in mouse xenografts. Furthermore, recent meta-analysis has also indicated that ALDH1A1 can serve as a predictor of poor prognosis in breast cancer patients [126]. However, although ALDH1 appears to be a good independent biomarker for early metastasis and poor survival in breast cancer, debate still exists. For example, the expression of ALDH1 is low in normal MaSCs, but highly expressed in luminal progenitors, which raises the question of whether ALDH1 can truly identify BCSCs [127]. Thus, the significance of ALDH1 as a biomarker of BCSCs has yet to be completely elucidated.

Overlapping tumorigenic populations have been observed in BCSCs isolated and enriched by different approaches. For instance, although there is only a small overlap between CD44^+^CD24^-/low^ and ALDH^+^ cells, ALDH^+^CD44^+^ cells seem to be more tumorigenic than cells that express one marker alone [16]. Although xenograft-initiating cells exist in both CD44^+^CD24^-^ and CD44^+^CD24^+^ cell populations, BCSCs are more highly enriched using the combinatorial marker profile CD44^+^CD49f^high^CD133/2^high^ [128].

## THERAPEUTIC PERSPECTIVES TARGETING BCSCS

Compared with common cancer cells, CSCs are slow-differential and have a lower ability to undergo apoptosis and a higher capability of DNA repair, making them more resistant to traditional chemotherapy and radiation cancer treatment [129]. Therefore, CSCs are regarded as the possible cause of therapy resistance and cancer recurrence. Although therapeutic methods targeting CSCs are widely studied and well-established, there is an alternative point of view in regards to such cells. Some researchers believe that proliferating cells, not CSCs, determine the progression, prognosis, resistance, and recurrence of advanced cancers that respond poorly to therapy [130]. Therapy-resistant clone cells, also known as cancer stemloids or stem cell-like cells, are proliferating, self-renewing cancer cells [131]. Therapeutic failure is often the result of the non-elimination of cancer stemloids. Therapy kills sensitive cells, resulting in the selection of resistant cells and the accumulation of stemness and resistance-conferring mutations [130, 131]. Thus, cancer stemloids are considered to be crucial targets for cancer therapy.

Although the application of CSCs in cancer therapy remains a topic of debate, we have focused on the therapeutic perspectives targeting BCSCs in this review. Considerable evidence has shown that BCSCs are responsible for the initiation, maintenance, metastasis, and recurrence of cancer, as well as resistance to traditional cancer treatment [129]. Recent reports using single-cell analysis have suggested that stem-like breast cancer cells initiate and propagate metastatic tumors [132]. Thus, targeting BCSCs is considered good clinical practice in the treatment of breast cancer, and can be achieved by a number of approaches, including chemotherapy sensitization of BCSCs, differentiating therapy, stem cell elimination, and suppression of regulatory pathways involved in self-renewal. For enhancement of chemotherapy sensitization, several pathways have been involved in sensitizing BCSCs, including increasing efflux of chemotherapeutic agents by the upregulation of cell-surface transporters of the ATP-binding cassette (ABC) family, increasing sensitivity to apoptosis though the alterations in the expression of Bcl2 family members, and reducing topoisomerase II expression [133, 134]. For targeting stem cell elimination, tumor formation is also driven by the expression of some genes involved in “stemness”, including Oct4, Nanog, and Sox2 [135]. Reversal of the expression of these genes might be a novel way to target BCSCs [129]. As BCSCs are often quiescent, they are also often resistant to traditional treatment. Differentiated BCSCs are easier to eliminate by differentiating therapy, which suggests that small molecules such as retinoic acid and other vitamin A analogues [136] can be used for breast cancer therapy treatment by differentiation induction. The *Retinal Determination Gene Network* (RDGN) is a regulatory network that is dysregulated in cancer [137, 138]. Key RDGN members, including DACH, EYA, and SIX, are potential therapeutic targets. In breast cancer, EYA raises tumor growth and increases the ratio of BCSCs [139]. SIX is enriched in the CD44^+^CD24^-/low^ subpopulation [140], whereas DACH might act as a tumor suppressor to reduce the number of BCSC subpopulations *in vitro* and *in vivo* though phosphorylating GSK3β and inhibiting Wnt signaling, compliance with findings in colorectal carcinomas [141, 142]. In this respect, research has also shown that the decreasing of DACH1 is tightly correlated with poor prognosis in basal-like breast cancer, suggesting the role of DACH1 as a potential predictor of survival in breast cancer patients [143, 144].

For suppression of regulatory pathways involved in self-renewal, the famous Notch, Hh, and Wnt signaling pathways have essential roles in the self-renewal of CSC populations. Here, we introduce some agents targeting these pathways and BCSCs in clinical trials (Table 1). Notch signaling influences self-renewal and lineage-specific differentiation of MaSCs [145], with Notch4 activity up regulated in BCSCs. Therefore, inhibition of Notch4 activity can decrease the BCSC population, and suppress tumor initiation [146]. The γ-secretase inhibitors (GSIs), targeting the Notch pathway, can inhibit the final proteolytic cleavage of Notch receptors, and result in suppression of the release of active intracellular fragments [147]. GSIs were the first Notch inhibitor developed for clinical cancer treatment [147], and exhibit anti-CSC activity in *ex vivo* patient-derived tumor specimens and breast-cancer-derived secondary mammospheres [148–151]. Notch pathway inhibitors combined with chemotherapy or other targeted agents exhibit strong anti-tumor activity. For HER2 positive subtype, GSIs together with trastuzumab completely cure and abrogate recurrence of tumor in mice [152]. In addition, optimal efficacy has been observed when Notch pathway inhibitors are combined with HER2 inhibitors in HER2-positive breast cancer [152, 153], with endocrine therapy in ER-positive breast cancer [154], and with taxanes and MET inhibitors in triple-negative breast cancer [155, 156].

**Table 1:** Investigational agent targeting BCSCs in clinical development *

Compound and combination or intervention	phase	Tumor type	clinicaltrials.gov identifier	status
**R04929097(GSI; Roche)**				
Plus vismodegib	I	Breast cancer (HER2−, metastatic or unresectable)	NCT01071564	Terminated
Plus letrozole	I	Breast cancer (postmenopausal ER+ stage II–III)	NCT01208441	Terminated
Plus carboplatin and paclitaxel before surgery	I	Stage II–III TNBC	NCT01238133	Terminated
Plus exemestane	I/II	Breast cancer (pre/postmenopausal, advanced-stage or metastatic)	NCT01149356	Terminated
Single agent	II	TNBC (advanced-stage, metastatic or recurrent)	NCT01151449	Terminated
**MK-0752 (GSI)**				
Plus Docetaxel	I/II	Metastatic Breast Cancer	NCT00645333	completed
**Vismodegib (Genentech)**				
With RO4929097 (Notch inhibitor)	I	Breast cancer	NCT01071564	Terminated
**Sonidegib (aka erismodegib and LDE225; Novartis)**				
Single agent	Randomized II	Breast cancer (stage II–III, ER−, HER2−)	NCT01757327	Withdrawn
**LGK-974 (Porcupine inhibitor; Novartis)**				
Single agent	I	breast neoplasms, TNBC	NCT01351103	Suspended
**Vantictumab (aka OMP-18R5; anti-Frizzled-1/2/5/7/8 antibody; OncoMed/Cellgene)**				
With paclitaxel	I	Breast cancer	NCT01973309	Recruiting
**CD105/Yb-1/SOX2/CDH3/MDM2-polyepitope Plasmid DNA Vaccine**				
Single agent	I	Breast cancer	NCT02157051	Recruiting

* Data are from ClinicalTrials.gov as of Augest 20, 2016; GSI, γ-secretase inhibitor; ER, oestrogen receptor; TNBC; triple-negative breast cancer.

The Wnt signaling pathway is important for the regulation and sustain of CSC properties. Abnormal Wnt/β-catenin signaling has also been reported in breast cancer [29, 33]. So far, several kinds of Wnt-signaling inhibitors have been developed for use in anti-cancer therapies. The LGK-974 drug, which inhibits porcupine and thereby decreases the secretion of Wnt proteins, is being tested in phase I trials in Wnt-ligand-dependent tumors, including breast cancer, melanoma, and pancreatic cancer [147]. Abnormal regulation of the Hh pathway is associated with many human malignancies. Several agents targeting the Hh pathway have been investigated in phase I and II clinical trials [157]. In addition, dysregulation of the Hh pathway is also reportedly involved in breast malignancies [158]. Of note, the PI3K/Akt pathway, which suppresses GSK3β, can concurrently activate the Wnt/β-catenin and Hh pathways [159]. In tamoxifen-resistant breast cancer cells, a suppressor of the PI3K/Akt pathway has been found to block both Hh and Wnt signaling, thereby showing better anti-tumor activity [159].
